# Identity and inequality misperceptions, demographic determinants and efficacy of corrective measures

**DOI:** 10.1038/s41598-024-62046-7

**Published:** 2024-05-29

**Authors:** K. Peren Arin, Deni Mazrekaj, Marcel Thum, Juan A. Lacomba, Francisco Lagos

**Affiliations:** 1 Zayed University, Abu Dhabi, United Arab Emirates; 2 Centre for Applied Macroeconomic Analysis, Canberra, Australia; 3 University of Granada, Granada, Spain; 4 Utrecht University, Utrecht, The Netherlands; 5 University of Oxford, Oxford, UK; 6 KU Leuven, Leuven, Belgium; 7 TU Dresden, Dresden, Germany; 8 ifo Dresden, Dresden, Germany; 9 CESifo, Munich, Germany

**Keywords:** Misinformation, Perception bias, Immigration, Poverty, Identity, Inequality, COVID-19, Information treatment, Health occupations, Health care

## Abstract

By conducting two waves of large-scale surveys in the United Kingdom and Germany, we investigate the determinants of identity and inequality misperceptions. We first show that people substantially overestimate the share of immigrants, Muslims, people under the poverty line, and the income share of the richest. Moreover, women, lower-income, and lower-educated respondents generally have higher misperceptions. Only income share misperceptions are associated more with people who place themselves on the left of the political spectrum. In contrast, the other three misperceptions are more prevalent among those who place themselves to the right. We then attempt to correct misperceptions by conducting a classic controlled experiment. Specifically, we randomly assign respondents into a treatment group informed about their initial misperceptions and a control group left uninformed. Our results indicate that information treatments had some corrective effects on misperceptions in Germany but were ineffective in the United Kingdom. Moreover, information treatments in Germany were more effective for men, centrists, and highly educated respondents. There is also no evidence of spill-over effects: correcting one misperception does not have corrective effects for the other misperceptions.

## Introduction

Misperceptions have significant implications across social, economic, and scientific realms, often leading to flawed policy decisions and hindering consensus. Debates surrounding issues such as vaccine administration, environmental policy, and immigration are particularly susceptible to the influence of misperceptions^1–3^. For instance, misperceptions regarding immigration have fueled support for anti-immigration policies in Europe, with voters demonstrating significant misconceptions about the impact of immigration^4,5^. Similarly, in the United States, misperceptions about becoming a minority have influenced electoral outcomes, contributing to the rise of polarizing political movements^6,7^. Additionally, studies highlight widespread ignorance and misperceptions regarding income distribution, which can shape individual policy preferences and voting behavior as well^8^. Despite race-based economic inequality being a longstanding issue in the US, Americans tend to overestimate progress toward racial economic equality, especially among white individuals^9^. Understanding the demographic determinants of these misperceptions is crucial for predicting societal attitudes and guiding policy-making processes.

Our primary goal is to provide a more comprehensive understanding of misperceptions across different societal domains. Whereas the previous studies have focused on a single topic, this study aims to examine misperceptions on four key domains: immigration, the Muslim population, poverty, and income distribution. We strive to identify common and unique socioeconomic determinants of the different misperceptions. In addition, we seek to better understand how providing information can correct misperceptions in different areas. Following Rodrik^10^, we chose these four particular misperceptions that are related to two topics populist parties usually exploit: identity and inequality. Whereas right-wing populist politicians mainly emphasize a cultural division (i.e., the national, ethnic, religious, or cultural identity of the “people” against outside groups), left-wing populists emphasize the economic division (i.e., wealthy groups who control the economy versus lower-income groups without access to power). We conducted two large-scale surveys in Germany (1813 respondents) and the United Kingdom (1259 respondents) in two waves. The first wave of the surveys was conducted between March 3, 2020, and March 30, 2020. The second wave of surveys was conducted almost 2 years later, between December 10, 2021, and December 14, 2021, for the same group of respondents. We quantified misperceptions by asking respondents to guess the share of immigrants, Muslims, people below the poverty line, and the income share of the wealthiest 10%. We subsequently compared the respondents’ guesses with the official statistics in each country to quantify the magnitude of misperceptions^1^. The numerical values for misperceptions allowed us to explore which individual socioeconomic characteristics determine misperceptions. In addition, we conducted a classic controlled experiment (also known as the pre-test/post-test control group design) to determine whether providing information to respondents about their misperceptions can affect their revised estimations. We randomly assigned about one-third of the respondents in each country to a treatment group that received information on their immigration misperceptions; another one-third of the respondents received information on their income misperceptions, and another one-third received no information. The information was of the form: *“In the last survey, you estimated population share of immigrants as 25%. In 2018, the population share of immigrants in the United Kingdom was 14%.”* In the first wave, we measured misperceptions of both the control and the treatment groups without providing any information on the actual number. In the second wave, we gave information only to respondents in the randomly assigned treatment groups.

The literature on misperceptions spans various disciplines, including psychology, sociology, economics, and communication and media studies. To guide our investigation, we formulated three hypotheses from this extensive literature.

### Hypothesis 1

We expect higher *identity* misperceptions among the less educated and younger populations. By contrast, the relationship between age and education on the one hand, and *inequality* misperceptions on the other is ambiguous.

Recent studies challenge previous explanations attributing biases in demographic estimation solely to factors like media bias or xenophobia, showing that identity misperceptions are better explained by the psychology of how people estimate quantities in general rather than attitudes toward particular groups^11,12^. The cognitive system’s tendency to prioritize uncommon and unexpected stimuli leads individuals to overestimate the prevalence of minority groups, creating an illusion of diversity^13^. Nevertheless, various studies indicate that older individuals, those with higher levels of education, men, and rural populations tend to have lower misperceptions of immigration^14–17^. Additionally, unpartnered individuals and members of ethnic minorities exhibit higher levels of misperceptions regarding immigrant populations^18^. Similarly, studies focusing on misperceptions about the size of the African American population in the US highlight women, less educated individuals, and younger adults as groups with higher levels of misperceptions^19^. Studies on inequality misperceptions reveal mixed findings regarding the impact of education and age. Some studies report that women and individuals with lower levels of education are more likely to misperceive their *social status*^15,20^. In contrast to the findings on identity misperceptions, the bias related to social status increases with age. The misperception of *income inequality* decreases with education and age^21^, contrary to other findings, highlighting the complexity of the relationship between education, age, and misperceptions. Similar mixed results exist for political orientation. While some argued individuals with a left-leaning political orientation tend to have less overestimation of their status^20^, others found no correlation between political orientation and biased income perceptions^15^.

### Hypothesis 2

a) During the pandemic, we expect an increase in both identity and inequality misperceptions. b) During the pandemic, we expect the increase in misperceptions to be smaller in Germany compared to the UK.

To curb the infection rate of the coronavirus, most countries implemented drastic measures such as lockdowns, and some countries even implemented curfews. Businesses deemed as “non-essential” were closed, and employees in these businesses were either laid off or furloughed. Some businesses went bankrupt. Although no previous studies have investigated how misperceptions evolved during the COVID-19 pandemic, several potential mechanisms suggest that identity misperceptions have likely increased. Previous studies indicate that poor economic conditions are a major driver of discrimination toward outside groups and minority populations. Analyzing 12 European countries, Semyonov et al.^22^ found that anti-foreigner sentiment is more pronounced in places with worse economic conditions. Similar findings were obtained by Kunovich^23^. The sociopsychological theory of discrimination explains these findings. Worsened economic conditions are often associated with more intensive labor market competition. Outside groups and minorities are often either blamed for the economic decline (scapegoats) or unfair economic competition (scabs)^24,25^. Given the worsened economic conditions, anti-foreigner sentiment may have grown. The sense of increased competition from an outgroup could magnify the perceived size of this group (*group threat theory*). As a result, we hypothesize that identity misperceptions regarding the share of immigrants and the Muslim population increased during the COVID-19 pandemic.

A similar line of argument can be drawn for inequality misperceptions. The COVID-19 pandemic has triggered a big shock for many businesses, which were completely shut down or could no longer operate profitably. This economic shock has stirred a debate about whether the pandemic will lead to more inequality and foster the existing trend toward higher inequality. However, actual inequality has hardly changed in Germany and the UK – probably also due to the dampening effects of the welfare state. The share of the top incomes remained roughly constant from 2018 to 2020/21, and poverty rates even fell slightly^26–28^. However, perceptions of the change in inequality may differ from the realized changes. Some particularly visible service sectors, such as the hotel and restaurant industry, had to close down for longer periods. Workers in these sectors had to stay home, had to reduce working hours, or may even have been laid off. Experiencing such setbacks in the neighborhood or hearing about them in the news may enlarge perceptions of inequality. At the other end of the income spectrum, a small elite also benefited from the shock^29^. The lockdowns limited the consumers’ opportunities (e.g., to shop at the most inexpensive location), created new scarcities, and increased profits in some businesses. Reports about the winners of the pandemic and rapidly increasing retail consumer prices may have contributed to enhanced misperceptions of inequality.

The policy response was swifter and more comprehensive in Germany than in the UK. Even before the pandemic, the per capita health spending in the United Kingdom was only two-thirds of Germany. In other words, Germany was better prepared with more intensive care beds and more widespread testing. During the pandemic, the German government’s response was much more immediate: the schools went into lockdown a week before the schools in the United Kingdom, and the German government swiftly introduced a fiscal stimulus program^30^. Therefore, the negative economic effects of the pandemic were much more pronounced in the U.K. The United Kingdom also suffered more COVID-related deaths than Germany, even though Germany has a much bigger population^31^. Hence, during the pandemic, we expect the increase in misperceptions to be smaller in Germany compared to the UK.

### Hypothesis 3

a) Information provision reduces misperceptions. b) Correcting one type of misperception will not reduce other misperception types.

Some previous papers on misperceptions investigated the consequences of providing information and thus correcting misperceptions, which may affect attitudes and policy stances. The evidence so far is inconclusive. Regarding identity misperceptions, some studies found that information provision cannot change people’s attitudes towards immigration^1,32^. Another study using survey experiments with more than 5000 university students in Germany shifted the students’ beliefs about refugees’ education through information provision. These beliefs significantly affected concerns about labor market competition but did not change general attitudes toward refugees^33^. Similarly, receiving information about the true share of immigrants led to more favorable attitudes towards immigrants but did not change the respondents’ policy preferences^34^.

Also, regarding inequality misperceptions, some studies found that providing information on the true degree of income inequality changes the redistributive preferences of respondents. The respondents were asked to assess their own relative position in the income distribution. After receiving information on their ranking within the income distribution, those who overestimated their relative position tended to favor more redistribution^14^, and those who underestimated their position wanted to reduce redistribution^15^. However, if respondents were not asked about their own position in the income scale but about their estimates of the general poverty rate, providing correct information had no effect on policy attitudes^35^. Only those participants who learned that they were net contributors to the tax-transfer system became more averse toward redistribution^16^.For a survey of to what extent correcting misperceptions influences beliefs and behaviors, see Hauser and Norton^36^.

In contrast to other papers, we do not test whether information provision changes attitudes. We aim to investigate to what extent an information treatment can correct misperceptions and whether it also leads to a rethinking of one’s estimates in other fields through cross-learning opportunities. Previously, this has only been investigated for COVID-19 misperception beliefs^37^. We expect that information treatment reduces misperceptions. Even if people do not recall the precise figure in the information treatment, they will recall that their previous estimates were too high. There might also be some Bayesian updating if people do not put 100 percent trust in the official figures we have provided^38,39^. Data interventions like ours were found to be more effective than narrative interventions in shifting discourse about racial wealth inequality and lowering estimates of Black-White wealth equality^40^. There is no reason a priori to expect different effects of the information treatments in Germany and the UK. To our knowledge, there are also no papers so far that analyze different types of misperceptions simultaneously. Hence, nothing is known about whether correcting one misperception also leads to rethinking estimates in other dimensions. The information treatment might shock the respondents, showing them how strongly their perceptions deviate from actual figures^41^. This might lead to a rethinking of one’s estimates in general. However, as we only correct misperceptions in one field, we do not expect that respondents recognize their tendencies to inflate estimates in all dimensions. It is unlikely that a single information treatment leads to a general recalibration of respondents’ estimates.

## Results

### Misperceptions are High

Table 1 shows descriptive statistics by country in wave 1. People significantly overestimate both the share of immigrants and the Muslim population. The actual share of immigrants is 14% in the UK and 17% in Germany. The respondents expected this share to be around 26% and 29%, respectively. Thus, the immigration misperception is about 12 percentage points in the two countries in our sample. The misperception is even larger when it comes to the share of the population of the Muslim faith. The respondents guessed that this share is about 21%, a substantial overestimation as the actual share is about 5% in both countries (i.e., the Islam misperception is about 16). The gap is also large even if respondents view Muslims as synonymous with those born in predominantly Muslim countries, as the total share of the foreign-born population is below these percentage levels.

**Table 1:** Descriptive Statistics (N = 3072).

	Germany				UK			
	Mean/Prop.	SD	Min.	Max.	Mean/Prop.	SD	Min.	Max.
Gender (1 is woman)	0.486				0.444			
Age								
18–35 y.o.	0.178				0.129			
36–54 y.o.	0.461				0.436			
55–70 y.o.	0.362				0.435			
Education (1 is high educated)	0.359				0.442			
Marital status (1 is married)	0.414				0.381			
Household income								
Low income	0.189				0.286			
Middle income	0.638				0.547			
High income	0.173				0.167			
Labour market position								
Employed	0.825				0.731			
Unemployed	0.018				0.027			
Out of labor force	0.157				0.242			
Political orientation								
Left	0.252				0.190			
Center	0.609				0.594			
Right	0.138				0.216			
Misperceptions immigration	12.318	21.583	−17.000	83.000	11.536	22.247	−14.000	86.000
Misperceptions Islam	16.251	15.045	−5.100	94.900	16.330	18.574	−4.400	95.600
Misperceptions poverty	6.660	19.651	−16.000	84.000	7.635	19.916	−15.700	84.300
Misperceptions income richest 10%	13.275	32.786	−25.700	74.300	19.536	33.230	−25.900	74.100
Observations	1813				1259			

The means on the misperceptions variables can be interpreted as follows: immigration misperceptions mean in Germany of 12.318 indicates that the respondents have overestimated the true share of immigrants in Germany by 12.318 percentage points. The number of observations varies for the different regressions on misperceptions due to missing estimates for misperceptions.

To capture inequality misperceptions, we asked the survey participants to estimate the share of people living below the poverty line and the income share of the richest 10% in their country of residence. These estimates were then contrasted with the most recent actual statistical figures. Table 1 shows that respondents also significantly overestimated inequality. The poverty rates amount to 16% in both countries. On average, the poverty rates were overestimated by about 7 percentage points in both countries. Likewise, the actual income share of the richest 10% is quite similar in the two countries, namely 26%. On average, the respondents overestimated those shares by 13 percentage points in Germany and 19.5 percentage points in the UK. Thus, it appears that respondents have high misperceptions in all dimensions.

### Determinants of misperceptions

We can also observe several determinants of misperceptions in Table 2. Identity misperceptions seem to go along with political orientation: in both countries, left-leaning respondents had smaller and right-leaning respondents had higher misperceptions than centrists. But even those who placed themselves on the left of the political spectrum significantly overestimated the share of immigrants and Muslims. Compared to centrists, their misperceptions are, on average, 2–6 percentage points smaller. One might argue that political orientation is also influenced by misperceptions, leading to a reverse-causality problem. By including political orientation in the regression, we follow the majority of the literature. Political orientation is mostly driven by long-term convictions, family traditions, and moral values. Even after controlling for political orientation, socioeconomic factors have additional explanatory power for the identity misperceptions. For instance, both immigration and Muslim misperceptions shrink with income and education. Moving from low to high education reduces misperceptions by 3–4.5 percentage points. The younger population exhibits larger misperceptions of the immigrant population. We also find that women tend to overestimate the share of immigrants and Muslims more than men.

**Table 2:** Misperceptions during the pandemic and determinants of misperceptions.

	Misperceptions Immigration		Misperceptions Islam		Misperceptions Poverty		Misperceptions Income	
	(1)	(2)	(3)	(4)	(5)	(6)	(7)	(8)
	Germany	UK	Germany	UK	Germany	UK	Germany	UK
Pandemic (ref: first wave)	−0.099	−1.089	−0.368	0.150	−1.953*	−1.830	1.596	−3.747
	(1.074)	(1.387)	(0.776)	(1.121)	(0.959)	(1.191)	(1.921)	(2.304)
Woman (ref: man)	4.522***	5.244***	5.179***	4.840***	5.220***	5.053***	−9.250***	−6.337**
	(1.077)	(1.412)	(0.762)	(1.163)	(0.946)	(1.242)	(1.949)	(2.363)
18–35 y.o. (ref: 36–54 y.o.)	3.864*	2.181	−1.429	−1.807	−0.333	−2.120	5.957*	−5.208
	(1.699)	(2.307)	(1.124)	(1.736)	(1.518)	(1.841)	(2.948)	(4.007)
55–70 y.o. (ref: 36–54 y.o.)	−1.146	−5.989***	0.928	−2.449+	−0.471	−2.834*	1.854	7.416**
	(1.255)	(1.569)	(0.889)	(1.298)	(1.104)	(1.376)	(2.229)	(2.634)
High educated (ref: low edu)	−4.329***	−4.548**	−2.993***	−3.365**	−4.155***	−3.113*	5.930**	6.063*
	(1.196)	(1.495)	(0.827)	(1.214)	(1.005)	(1.253)	(2.129)	(2.490)
Separated/single (ref: married/cohabiting)	−1.948	−1.852	−1.028	0.704	−3.266**	−0.304	1.008	3.361
	(1.277)	(1.424)	(0.900)	(1.215)	(1.087)	(1.286)	(2.181)	(2.526)
Middle income (ref: low)	−2.776+	−8.350***	−0.467	−5.953***	−6.608***	−10.076***	0.031	−2.354
	(1.611)	(1.712)	(1.211)	(1.493)	(1.543)	(1.637)	(2.899)	(3.041)
High income (ref: low)	−3.730+	−5.935*	−2.740+	−4.979*	−12.507***	−8.973***	11.946**	−6.825+
	(2.103)	(2.452)	(1.486)	(1.962)	(1.889)	(2.065)	(3.787)	(4.049)
Unemployed (ref: employed)	0.746	3.978	6.297+	−0.510	4.350	2.992	1.680	8.794
	(4.645)	(4.769)	(3.683)	(3.456)	(4.768)	(3.489)	(8.866)	(8.496)
Out of labor force (ref: employed)	−1.671	−0.365	0.330	0.293	−2.964*	0.534	−0.519	8.302**
	(1.413)	(1.664)	(1.071)	(1.450)	(1.261)	(1.510)	(2.736)	(2.875)
Left (ref: center)	−3.308*	−6.220***	−2.194*	−6.184***	−3.031**	−3.007+	8.475***	8.875**
	(1.295)	(1.808)	(0.867)	(1.212)	(1.102)	(1.573)	(2.318)	(3.284)
Right (ref: center)	3.701*	1.850	5.683***	2.704+	3.812*	−5.955***	1.450	−2.939
	(1.588)	(1.853)	(1.269)	(1.529)	(1.559)	(1.353)	(2.864)	(2.753)
Constant	14.373***	21.535***	15.659***	21.027***	14.379***	17.164***	9.051*	13.980***
	(2.108)	(2.252)	(1.551)	(1.873)	(1.941)	(2.080)	(3.853)	(3.823)
Observations	1,507	1,034	1,422	957	1,518	1,038	1,157	787
Adj. R-squared	0.036	0.070	0.069	0.068	0.083	0.099	0.064	0.062

All estimates are from linear models estimated by ordinary least squares.

Only respondents in the control group have been included.

Robust standard errors are in parentheses.

+$p<$.10, *$p<$.05, **$p<$.01, ***$p<$.001 (two-tailed tests).

When we turn to the socioeconomic determinants of inequality-misperceptions, political orientation, once again, plays an important role in both income distribution and poverty misperceptions. The majority of the previous literature reports that social proximity to a social group (e.g., the poor) leads to a stronger overestimation of the size of this group. In Germany, however, right-wing respondents overestimate poverty more than centrist and left-wing respondents. In the U.K., the centrists provide the largest overestimation of the poor. The misperceptions of the top incomes are stronger among left-leaning respondents. As for identity misperceptions, we find that the other socioeconomic variables have strong explanatory power for inequality misperceptions even when controlling for political orientation. In contrast to identity misperceptions, however, socioeconomic factors driving inequality misperceptions are less uniform. For instance, women show a stronger overestimation of the share of those under the poverty line; men exhibit a stronger overestimation of the income share of the richest. Highly educated respondents exhibit significantly smaller poverty misperceptions but significantly larger misperceptions of top incomes. In Germany, the same holds for high-income respondents. The distance or proximity to the rich and poor in society might matter for the magnitude of the misperceptions in the sense that those who are better off or better educated might strive for even higher income, focus on the top incomes, but exaggerate these income positions even more. Overall, Hypothesis 1 regarding the socioeconomic determinants of misperceptions is confirmed.

### Misperceptions and the COVID-19 pandemic

To capture the potential change in misperceptions during the COVID-19 pandemic, we use the *Pandemic* variable, which takes the value of 1 for the second wave of our survey. Table 2 indicates that most misperceptions were not altered during the pandemic, except for poverty misperceptions in Germany. Specifically, during the pandemic, poverty misperceptions have been reduced. The rest of the misperceptions remained stable and, therefore, relatively high. Hence, Hypothesis 2 is rejected.

Initially, there were many concerns regarding the increased inequality during the pandemic. The government reacted with a large-scale subsidy program in Germany, giving financial assistance to companies and increasing the availability of short-time work for employees. With the ‘short-time work’ transfers, the German government replaced the labor income of employees if companies had to reduce the working hours due to a recession. During the COVID-19 pandemic, the German government increased the program so that workers on short-time work received between 70 and 87 percent of their regular salaries. This program significantly reduced the risk of higher inequality at the lower end of the income distribution. In April 2020, more than 6 million employees were on short-term work^42^. There were first analyses towards the end of the year 2020^43^ that the pandemic did not increase income inequality in Germany. The active response by the government, the personal experiences of fairly stable incomes on the employees’ side, and the massive media reports of stable disposable incomes may have reduced fears that a larger share of the population will be in poverty due to the pandemic. The experience that the insurance function of the German welfare state actually worked may have helped reduce the overestimation of poverty within the country.

### The effect of information treatments

To investigate whether information provision can correct misperceptions and whether this recalibration works similarly for all demographic groups, we implemented an information treatment in the survey. One-third of the respondents during the second wave were informed about the true share of the foreign-born population. We did the same for another one-third regarding the information on top incomes.

**Table 3:** The effect of information treatments.

	Misperceptions Immigration		Misperceptions Islam		Misperceptions Poverty		Misperceptions Income	
	(1)	(2)	(3)	(4)	(5)	(6)	(7)	(8)
	Germany	UK	Germany	UK	Germany	UK	Germany	UK
Second wave (ref: first wave)	−0.047	−1.205	−0.313	0.110	−1.874^∗^	−1.813	0.994	−3.875+
	(1.071)	(1.381)	(0.774)	(1.123)	(0.956)	(1.190)	(1.922)	(2.303)
Immigration treatment (ref: no treatment)	1.305	−0.943	−0.702	1.270	0.393	0.090	−2.080	2.060
	(1.200)	(1.506)	(0.829)	(1.309)	(1.090)	(1.344)	(1.995)	(2.454)
Income treatment (ref: no treatment)	1.535	−2.123	0.370	0.301	−0.685	−0.516	−0.364	−0.965
	(1.216)	(1.457)	(0.852)	(1.261)	(1.065)	(1.296)	(2.021)	(2.398)
Second wave × Immigration treatment	−3.315^∗^	−2.001	0.039	−2.177	0.356	−2.071	0.950	2.334
	(1.616)	(2.060)	(1.143)	(1.834)	(1.484)	(1.804)	(2.758)	(3.444)
Second wave x Income treatment	−2.730	0.521	−0.696	−2.032	0.603	0.433	−8.048^∗∗^	−3.790
	(1.682)	(2.070)	(1.227)	(1.708)	(1.458)	(1.750)	(2.664)	(3.217)
Woman (ref: man)	4.658^∗∗∗^	3.895^∗∗∗^	5.063^∗∗∗^	4.043^∗∗∗^	5.493^∗∗∗^	4.151^∗∗∗^	−9.132^∗∗∗^	−5.426^∗∗∗^
	(0.701)	(0.880)	(0.493)	(0.764)	(0.634)	(0.782)	(1.131)	(1.398)
18–35 y.o. (ref: 36–54 y.o.)	3.291^∗∗^	4.270^∗∗^	−1.875^∗∗^	2.245	−0.618	0.020	−0.217	−7.129^∗∗^
	(1.121)	(1.523)	(0.674)	(1.394)	(0.940)	(1.302)	(1.639)	(2.452)
55–70 y.o. (ref: 36–54 y.o.)	−1.761^∗^	−5.006^∗∗∗^	0.392	−1.654^∗^	−1.184+	−3.321^∗∗∗^	5.036^∗∗∗^	3.575^∗^
	(0.780)	(0.976)	(0.587)	(0.830)	(0.700)	(0.846)	(1.294)	(1.539)
High educated (ref: low edu)	−3.014^∗∗∗^	−4.943^∗∗∗^	−2.796^∗∗∗^	−4.831^∗∗∗^	−4.841^∗∗∗^	−4.779^∗∗∗^	6.488^∗∗∗^	3.264^∗^
	(0.735)	(0.871)	(0.517)	(0.759)	(0.617)	(0.754)	(1.190)	(1.420)
Separated/single (ref: married/cohabiting)	−1.117	−1.018	−0.524	0.414	−2.188^∗∗^	−0.607	1.936	3.819^∗^
	(0.822)	(0.920)	(0.562)	(0.835)	(0.698)	(0.830)	(1.275)	(1.505)
Middle income (ref: low)	−2.214^∗^	−3.901^∗∗∗^	−1.559^∗^	−2.949^∗∗^	−6.333^∗∗∗^	−6.863^∗∗∗^	−1.370	−1.001
	(1.047)	(1.087)	(0.787)	(0.997)	(1.041)	(0.997)	(1.716)	(1.792)
High income (ref: low)	−4.615^∗∗∗^	−2.009	−3.336^∗∗∗^	−2.482^∗^	−12.630^∗∗∗^	−6.646^∗∗∗^	4.782^∗^	−0.913
	(1.340)	(1.427)	(0.958)	(1.245)	(1.214)	(1.251)	(2.222)	(2.288)
Unemployed (ref: employed)	1.789	3.606	2.944	0.515	4.239	3.450	2.598	3.972
	(2.901)	(2.860)	(2.229)	(2.534)	(3.062)	(2.705)	(5.322)	(5.372)
Out of labor force (ref: employed)	−1.920^∗^	0.480	−0.900	1.827+	−2.314^∗∗^	0.064	1.469	6.970^∗∗∗^
	(0.926)	(1.050)	(0.686)	(0.947)	(0.840)	(0.923)	(1.634)	(1.715)
Left (ref: center)	−2.944^∗∗∗^	−6.953^∗∗∗^	−2.494^∗∗∗^	−5.851^∗∗∗^	−1.330+	0.141	4.920^∗∗∗^	11.027^∗∗∗^
	(0.831)	(1.019)	(0.573)	(0.820)	(0.733)	(0.932)	(1.354)	(1.825)
Right (ref: center)	3.669^∗∗∗^	1.830	4.691^∗∗∗^	0.934	2.540^∗∗^	−3.719^∗∗∗^	0.729	−4.054^∗^
	(1.015)	(1.141)	(0.778)	(0.979)	(0.967)	(0.886)	(1.621)	(1.651)
Constant	13.554^∗∗∗^	18.168^∗∗∗^	17.004^∗∗∗^	19.081^∗∗∗^	13.771^∗∗∗^	15.265^∗∗∗^	11.486^∗∗∗^	15.337^∗∗∗^
	(1.498)	(1.642)	(1.072)	(1.388)	(1.420)	(1.480)	(2.505)	(2.671)
Observations	3,586	2,477	3,387	2,281	3,589	2,482	3,156	2,157
Adj. R-squared	0.040	0.064	0.065	0.060	0.087	0.075	0.050	0.062

All estimates are from linear models estimated by ordinary least squares.

Robust standard errors are in parentheses.

+$p<$.10, ^∗^ $p<$.05, ^∗∗^ $p<$.01, ^∗∗∗^ $p<$.001 (two-tailed tests).

The interaction term between the treatments in Table 3 and the second wave reveals some insights. In the United Kingdom, the information treatments were completely ineffective. In Germany, on the contrary, information treatments worked to some extent. More specifically, those who received information on the true share of the foreign-born population revised their excessively high estimates downwards. Those who received the information regarding top income share also recalibrated their estimates and moved toward the actual figures. Information treatments in one domain have no spill-over effects on other domains. The only significant effect of the information treatments is within the domain in which the correction was provided.

**Figure 1:**
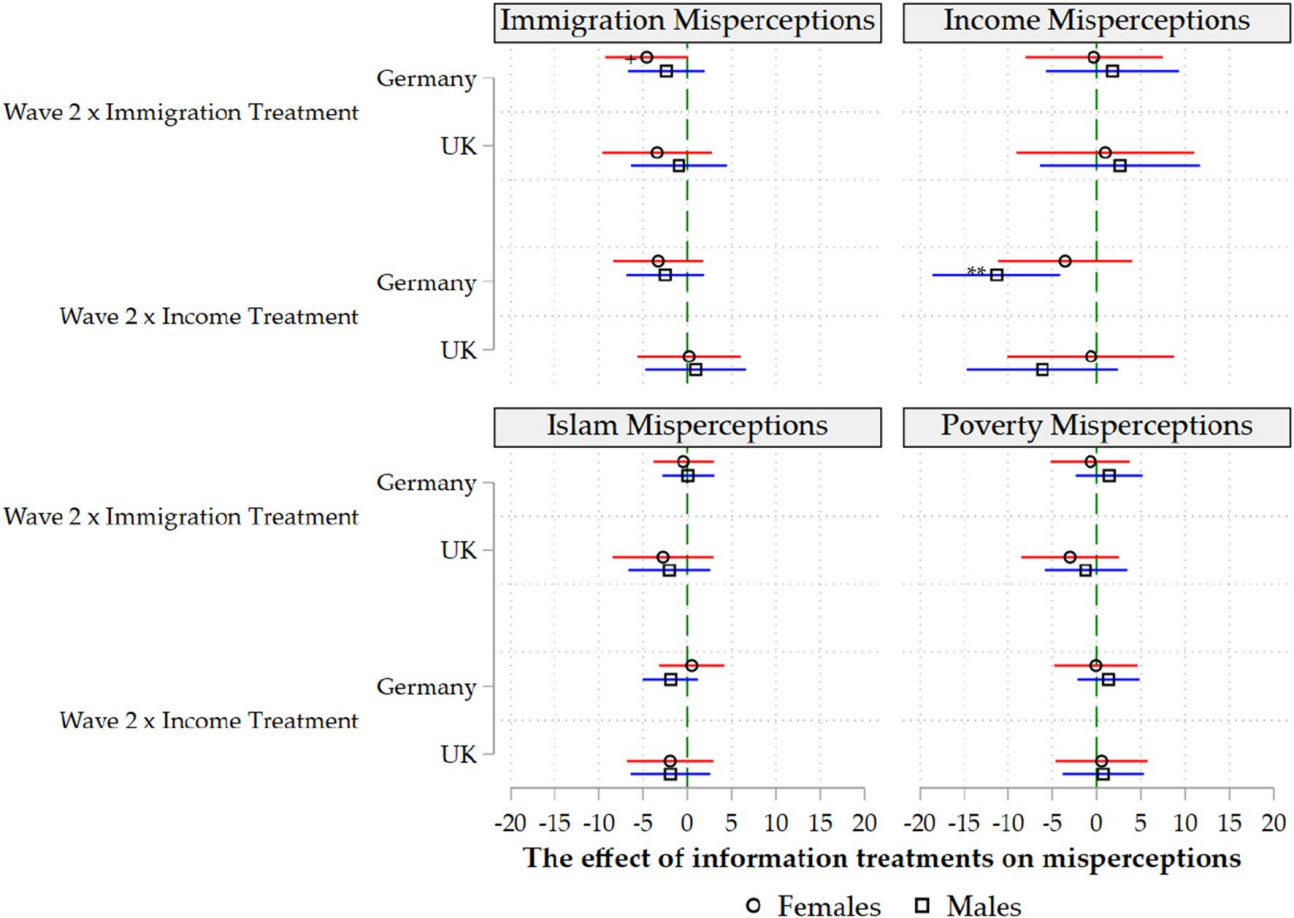
*Note* All estimates are have been estimated by ordinary least squares using robust standard errors and with control variables and main effects included. The results for the interaction terms as in Eq. (2) are reported. +$p<$.10, *$p<$.05, **$p<$.01, ***$p<$.001 (two-tailed tests).

**Figure 2:**
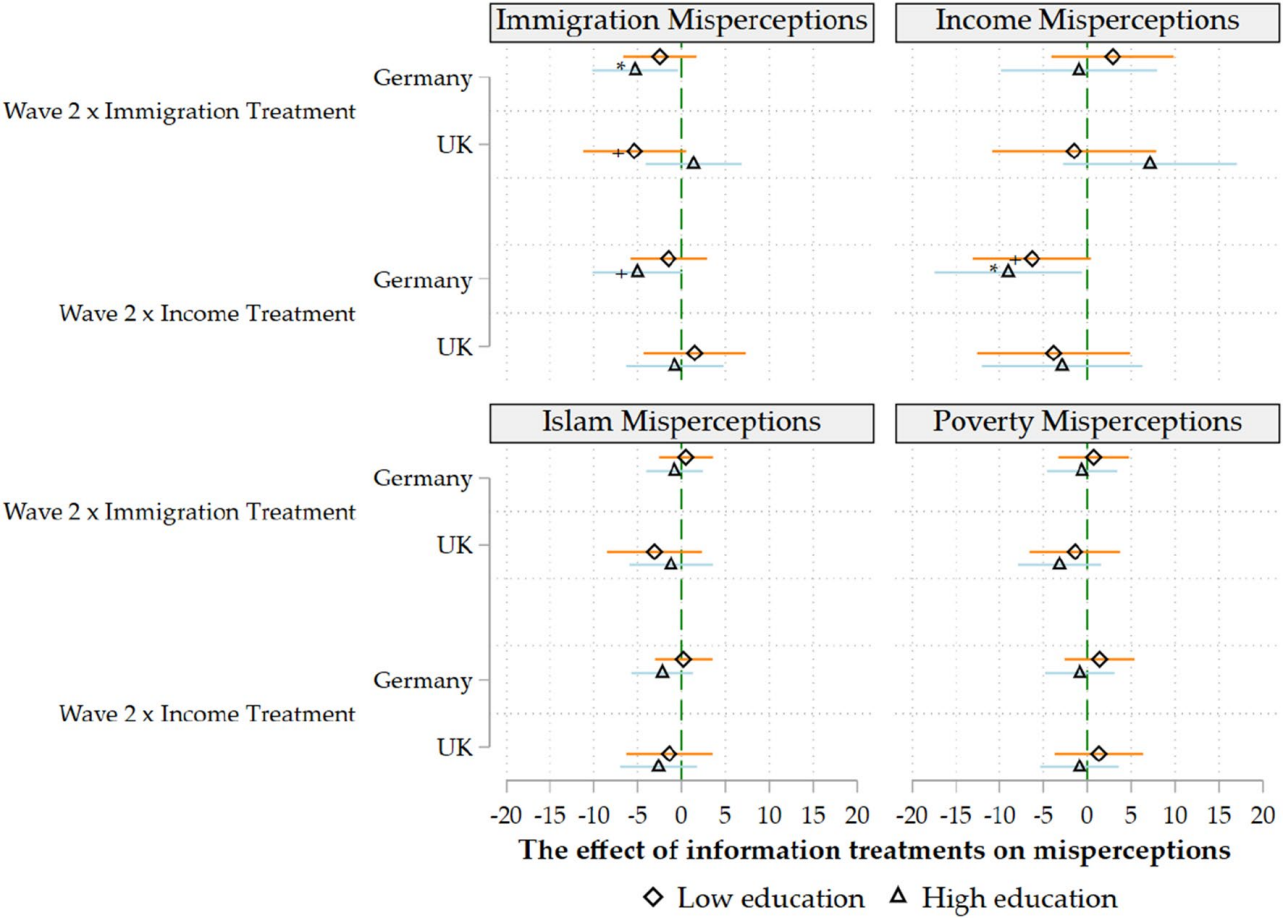
*Note* All estimates are have been estimated by ordinary least squares using robust standard errors and with control variables and main effects included. The results for the interaction terms as in Eq. (2) are reported. +$p<$.10, *$p<$.05, **$p<$.01, ***$p<$.001 (two-tailed tests).

**Figure 3:**
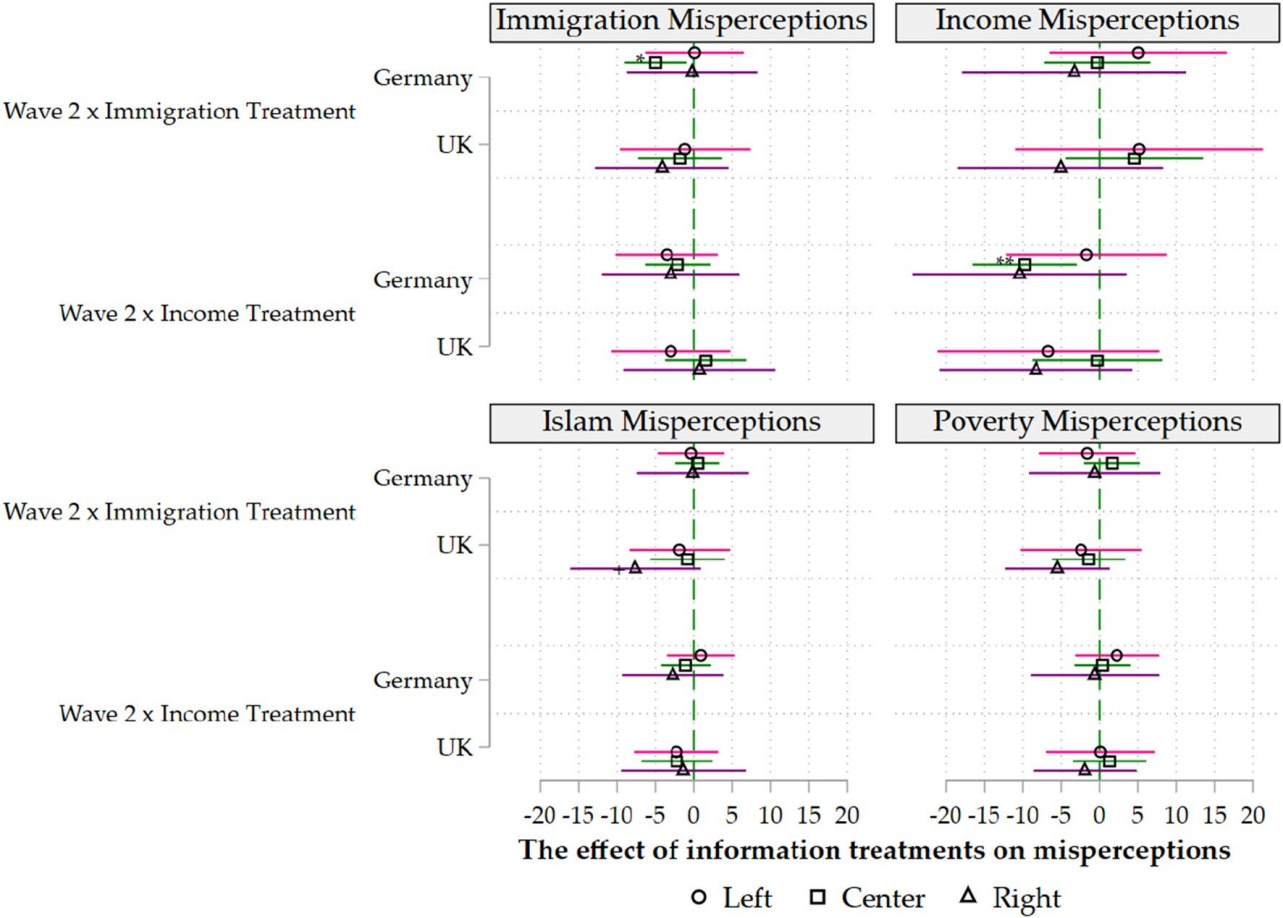
Misperceptions by Country and Political Orientation. Notes. All estimates are have been estimated by ordinary least squares using robust standard errors and with control variables and main effects included. The results for the interaction terms as in Eq. (2) are reported. +$p<$.10, *$p<$.05, **$p<$.01, ***$p<$.001 (two-tailed tests).

In the next three models, we investigate the socioeconomic determinants of the information treatments’ effectiveness (Figs. 1, 2 and 3). Our results show that the previous results for Germany are driven by highly educated, centrist men. The regression coefficients become statistically insignificant within split samples for women, the low-educated and those who consider themselves “extreme-right” or “extreme-left”.

The results of the information treatments partially confirm Hypothesis 3. The information treatments reduced misperceptions significantly in Germany, but had no effect in the UK (Hypothesis 3a is partially confirmed). Moreover, we do not find spillovers of an information treatment on other misperceptions, which confirms Hypothesis 3b.

## Discussion

We constructed a novel large dataset on identity and inequality misperceptions to provide a more comprehensive understanding of misperceptions across different societal domains. We document that respondents have large misperceptions in all four dimensions studied. We also observed that during the COVID-19 pandemic, there has not been a significant change in the magnitude of misperceptions, with only one exception: the overestimation of poverty was reduced in Germany during the pandemic. The overestimation of immigration aligns with the existing literature^1^.

We also presented evidence to identify which individual-level socioeconomic determinants can explain misperceptions. Even after controlling for political orientation, consistent with our a priori expectations, we document that women and low-educated individuals tend to have the largest misperceptions. However, the overestimation of top incomes is larger among men and the highly educated. Our findings regarding immigration are in line with several studies, which also identify low education and gender (women) as socioeconomic factors significantly driving higher misperceptions^1,17,18,24^. The same socioeconomic characteristics proved significant for the overestimation bias in the studies on perceptions of US minorities^19,44^. There is one major difference to some other studies^1,24^. Namely, we find that identity misperceptions increase when respondents move from left to right in the political spectrum. By contrast, in previous studies, left- and right-wing respondents did not exhibit statistically significant differences in their perception of the share of immigrants. Misperceptions were even smaller among politically conservative respondents^18^.

Finally, we aimed to understand whether providing information can correct misperceptions in different areas. Our results showed that information treatments were slightly effective in Germany. A closer look into the data revealed that this result is driven by men, centrists, and the highly educated.

We contribute to the literature in several ways. First and foremost, previous literature provides some socioeconomic determinants for several misperceptions, but not within a unified framework^1,45^. We add to this literature by simultaneously investigating misperceptions in several dimensions. This allows us to gain a more comprehensive understanding of the broader landscape of misinformation. Our analysis provides additional insights into the interconnectedness of misperceptions and the common underlying causes. Second, during our survey period, the COVID-19 pandemic started spreading rapidly; the World Health Organization (WHO) declared a public health emergency of international concern, and nationwide lockdowns as well as school closures were implemented in both countries^46^. Germany and the United Kingdom approached the global pandemic differently, offering a unique opportunity to observe how the pandemic and the different government responses influenced misperceptions. Investigating how the pandemic relates to misperceptions in different countries allows for identifying contextual factors that influence public perceptions and behaviors.

Our final contribution is conducting a classic controlled experiment (also known as the pre-test/post-test control group design) to determine whether providing information to respondents about their misperceptions can affect their revised estimates. In this respect, we contribute to the literature on the effectiveness of the information provision. Bursztyn and Yang^41^ argued that *“Experimental treatments to re-calibrate misperceptions generally work as intended; they sometimes lead to meaningful behavior changes.*” However, very little is known under which conditions and for which population subgroups the information treatments work as intended. Some studies^34^ have shown that people do not often revise their policy stances when provided with correct information. We document that certain individuals do not even revise their estimates when provided hard-fact statistical information. Our setting with several dimensions of misperceptions also allowed us to investigate whether correcting misperceptions in one domain (e.g., immigration) leads to reduced misperceptions in another domain (e.g., poverty) due to questioning one’s beliefs and cross-learning opportunities. We found that this was not the case. In sum, the study provides valuable insights for policymakers by elucidating the conditions under which information treatments work as intended and exploring potential spillover effects across different domains of misperceptions.

There are also some limitations of our survey approach. First, the respondents’ true estimates might deviate from the numbers they gave us. Respondents might give what they perceive to be socially desirable answers; this might distort the perception biases downward. We only paid them to complete the entire survey but did not specifically incentivize them to complete the questions regarding the misperceptions. Second, it was documented by the previous literature that many people cannot easily translate their perceptions into numerical terms^44^. Third, we would have liked to enhance the treatment side of our study. In addition to correcting the biases in the estimated numbers, it would be interesting to know whether treatments lead to changes in attitudes or policy stances. This would have required additional questions on policy stances and additional treatments, e.g., by providing information on emotional aspects of immigration or poverty. Fourth, we do not have a perfectly controlled panel of respondents. In particular, the number of respondents decreased from the first to the second wave. This is partly due to the permanent change in participation in online panels and can hardly be avoided. Fifth, a survey covering more countries would be desirable as our survey has shown that there are differences between Germany and the UK.

Even though misperceptions may have no direct impact on policy choices, they may have an impact on social outcomes. For instance, revealing stereotypes may help decrease discrimination in the context of teachers’ bias in grading immigrant children and thus positively contribute to their educational outcomes^47^. As our study provides evidence for the difficulty of correcting misperceptions, future research may investigate why certain people do not recalibrate their estimates.

## Data

### Data collection

We conducted large-scale surveys in Germany and the United Kingdom. The survey was designed and programmed by the authors via Qualtrics, and was provided to the respondents in their native languages. The first wave of the survey was conducted between 3 March 2020 and 30 March 2020, and the second wave was between 10 December 2021 and 14 December 2021. Both waves were administered by the company Respondi (https://www.respondi.com/EN/), which has access to representative samples of respondents to whom they send out survey links by email. Zayed University Research Ethics Committee approved all survey questions. The survey was carried out in accordance with relevant guidelines and regulations. Informed consent was obtained from all participants who were 18 years or older.

In the first wave, we applied quotas on gender, age, income, and labor market status to create a representative survey^48,49^. Once the quota had been met, the respondents were no longer permitted to submit a response. In the second wave, all respondents who remained in Respondi’s panel were contacted. The summary statistics for each wave, alongside population values, are presented in Table 4. The sample in the first wave appears to be rather representative of the population with some differences. Namely, there appear to be fewer people with a high income in the United Kingdom, and married or cohabiting people are underrepresented in both countries. In the second wave, we contacted all the people from wave one and obtained a response rate of about 36% in Germany and 28% in the UK. A notable difference between the first and the second wave is that people between 18 and 35 are underrepresented in the second wave. The remaining characteristics appear to be rather consistent with the first wave.

**Table 4:** Comparing sample statistics and population statistics.

	Germany			UK		
	Population	1st wave	2nd wave	Population	1st wave	2nd wave
Woman	49.6	53.5	48.6	50.3	50.7	44.6
18–35y.o.	31.3	30.0	14.4	35.1	32.5	10.2
36–54y.o.	37.0	39.9	45.3	37.5	36.7	42.1
55–70y.o.	31.7	30.1	40.3	27.4	30.8	47.7
High educated	28.0	34.1	38.7	43.3	42.8	45.1
Married/cohabiting	61.1	42.7	40.9	63.1	40.7	38.8
Low income	24.4	21.3	16.6	27.0	29.2	25.3
Middle income	59.4	63.8	63.3	43.0	54.9	54.2
High income	16.3	14.9	20.1	30.0	15.9	20.4
Employed	75.4	77.5	78.6	75.3	72.8	70.7
Unemployed	2.3	2.1	1.6	2.4	4.6	2.8
Out of labor force	22.2	20.4	19.8	22.3	22.6	27.5
Left		26.9	23.7	22.1	20.0	19.5
Center		58.7	62.2	63.4	56.6	59.4
Right		14.4	14.2	14.5	23.4	21.1
Observations		5032	1813		4450	1259

This table shows summary statistics from our sample alongside representative statistics of the population in each country. The numbers in the table represent percentages. Data for gender, age, employed, household type and unemployed come from Eurostat. Eurostat is the statistical office of the European Union: https://ec.europa.eu/eurostat/. “Married/cohabiting” captures the share of the adult population living as a couple; the data for the entire population is taken from the Labor Force Statistics ($LFST \_ HHNHTYCH$, number of private households by household composition). The education data also comes from the Labor Force Survey ($LFSA \_ PGAED$, population by sex, age and educational attainment level) and refers to the population aged 20-64. For income data the sources are: 1) For Germany: National Statistics Institute (https://www.destatis.de/DE/Home/_inhalt.html), income levels (monthly net household income) are: less than 1500€; 1500€–2999€; 3000€ or more; 2) For the United Kingdom: National Statistics Institute (https://www.gov.uk/search/research-and-statistics), income levels (gross weekly household income) are: less than £400; £400–£1000; £1000 or more. Employment data is taken from the Labor Force Survey (population by sex, age, citizenship and labour status, $LFSQ\_PGANWS$). Employed category also includes self-employed people.

The average time for completion of the survey was 21 min, and the respondents were paid only if they fully completed the survey. We removed respondents who did not complete the demographic part of the questionnaire and who completed the survey either very fast (in less than 5 min) or very slow (more than 2h). Our final sample includes 3072 respondents aged 18-70 who filled in the questionnaire in both waves and, therefore, 6144 observations. Not all respondents filled out all the questions that we used as outcomes. Therefore, we let the sample sizes vary by outcome.

### Variables construction

The survey has two components: (1) socioeconomic characteristics and (2) misperceptions. The complete English version of the survey is provided in the Online Supplement.

In the first set of questions, respondents were asked about socioeconomic characteristics. To enable comparisons with the previous literature, we followed Alesina et al.^1^ and constructed the following socioeconomic characteristics: gender (1 is woman, 0 is man), age (18–35 years old, 36–54 years old, and 55–70 years old), education (1 is high-educated, 0 is low-educated), marital status (0 is married or cohabiting, 1 is separated or single), household income (low-income, middle- income, high-income), labor market position (employed, unemployed, out of the labor force), and political orientation (left, center, right). Education is defined as high-educated if the respondents finished tertiary education, and low-educated if otherwise. For Germany, household income is defined as low income if the net monthly household income is below 1500 EUR, middle income between 1500 EUR and 2999 EUR, and high income if the net monthly household income is above or equal to 3000 EUR. For the UK, the corresponding thresholds refer to gross weekly household incomes and are less than £400; £400–£1000; more than £1000. Political orientation is determined from the respondents’ answers to the question *“In politics, people sometimes talk about “left” and “right”. Please indicate on a scale of 0–10 where you would place yourself (0 = Left; 10 = Right).”*. We recategorized this variable for ease of interpretation to left (from 0 to 3), center (from 4 to 6), and right (from 7 to 10).

In another set of questions, we explored misperceptions. For identity misperceptions, we elicited the respondents’ perceptions of the number of immigrants and the number of people practicing Islam. The particular questions (for the United Kingdom) are as follows: i) Think about all of the people currently living in the United Kingdom. Out of every 100 people in the United Kingdom, how many are born in another country?, and ii) Fill in the boxes below to indicate how many out of every 100 people in the United Kingdom you think to practice Islam. For inequality misperceptions, participants were asked about their perceptions of poverty and the share in income of the richest people. The questions are as follows: i) Out of every 100 adult people born in the United Kingdom, how many live below the poverty line?, and ii) What do you think is the income share of the richest 10% of all people living in the United Kingdom? In the survey, we defined the poverty line as the estimated minimum level of income needed to secure the necessities of life. The dummy variable that takes the value of 1 for the second-wave responses and 0 for the first-wave responses is our *Pandemic* variable, which captures the later stages of the pandemic.

We operationalized misperceptions as indices that subtract the actual statistics to the respondents’ guesses. For instance, if the respondents guessed that there are 30 immigrants for every 100 people, whereas there are actually five immigrants for every 100 people in their country, the misperception index would amount to 25. Note that the misperception index can also be negative if people’s guesses are below the actual numbers. If a person guesses four and the actual number is 5, the misperception index would amount to −1. Thus, we consider four misperception indices as outcomes: misperception of the share of immigrants, o the share of the Muslim population, of the share of people below the poverty line, and of the income share of the richest 10%. We consistently used the 2018 data for the actual numbers. First, this was the official data that was available at the time of the interviews. Second, as discussed before, the actual numbers hardly changed over time.

The actual statistics were obtained from various sources as outlined in the Online Supplement. We gathered the most recent data available to the public in March 2020. The only data that was available in this period applied to 2018 for most indices. It could be argued that people may actually have a better perception of their environment than the official statistics from 2 years ago. In this case, our estimates of misperceptions would be overestimated. However, this is unlikely to be the case as most of these indicators, such as the number of immigrants or the income share of the top 10% are unlikely to fluctuate greatly in such a short period. For instance, recent statistics show that the share of the foreign-born population had remained constant throughout 2019 to 2021 in Germany and the UK, respectively.

When correcting misperceptions in the second wave, we used two treatments. We told the treated respondents for immigration misperceptions *“In the last survey, you estimated population share of immigrants as X%. In 2019, the population share of immigrants in Country Y was Z%.”* Analogously, we told the treated respondents for income misperceptions *“In the last survey, you estimated income share of the richest 10% as X%. In 2019, the income share of the richest 10% in the Country Y was Z%.”*. Respondents in the control group received no information. After this question, respondents were again asked about their perceptions of immigration, the Muslim population, poverty, and income inequality. We coded the treatment variable as an indicator with three categories, namely (0) no treatment, (1) immigration treatment, and (2) income treatment.

## Methodology

We estimate how misperceptions relate to different demographic characteristics and the COVID-19 pandemic using a linear model estimated by Ordinary Least Squares (OLS):

1 $$\begin{aligned} Y_{it} = \alpha + \beta W_{it} + \mathbf {\mu D_{it}} + \epsilon _{it} \end{aligned}$$

In Eq. (1), $Y_{it}$ represents the identity and inequality misperception indices for individual *i* in wave *t*. One variable of interest is the pandemic variable $W_{it}$, given a value of 1 in wave 2 and a value of 0 in wave 1. The parameter $\mathbf {\beta }$ represents the change in misperceptions in wave 2 with respect to wave 1. We also relate a vector of socioeconomic factors $\mathbf {D_{it}}$ to misperceptions. These include the gender (1 is woman, 0 is man), age (18–35 years old, 36–54 years old, and 55–70 years old), education (1 is high educated, 0 is low educated), marital status (0 is married or cohabiting, 1 is separated or single), household income (low income, middle income, high income), labor market position (employed, unemployed, out of the labor force), and political orientation (left, center, right). We perform all analyses by country, and in each specification, we use robust standard errors. Because we provide information treatments that may have influenced misperceptions (see below), we estimate Eq. (1) only for the sample of respondents who did not receive any information treatments. The reader should keep in mind that we are not able to account for endogeneity arising from omitted factors or reverse causality and, therefore do not make any causal claims. Nor is it possible to causally interpret multiple coefficients of the same model^50,51^. Nonetheless, we do provide timely evidence of how the COVID-19 pandemic is related to misperceptions.

We then conduct a classic controlled experiment, also known as a pre-test post-test control group design, to attempt to correct misperceptions. We randomized participants into three groups: a treatment group that received the immigration treatment in the second wave but not in the first wave, a treatment group that received the income treatment in the second wave but not in the first wave, and a control group that did not receive any treatments in both waves. The difference in the control group’s misperceptions from the first to the second wave indicates the change in misperceptions that could be expected to occur without exposure to information treatments. The difference in the treatment groups’ misperceptions from the first to the second wave indicates the change in misperceptions that could be expected to occur with exposure to the information treatments. As a result of the random assignment, the difference between the change in the treatment groups and the change in the control group is the amount of change in misperceptions that can be attributed solely to the influence of the information treatments. We formulate the empirical model as follows:

2 $$\begin{aligned} Y_{it} = \alpha + \beta W_{it} + \gamma Imm_{it} + \delta Inc_{it} + \theta (W_{it}*Imm_{it}) + \lambda (W_{it}*Inc_{it}) + \mathbf {\mu D_{it}} + \epsilon _{it} \end{aligned}$$

Apart from the parameters outlined in Eq. (1), Eq. (2) includes the information treatment variables and interaction terms. Namely, $Imm_{it}$ represents a dummy indicator for immigration treatment, and $Inc_{it}$ is a dummy indicator for income treatment. The parameters of interest are $\mathbf {\theta }$ and $\mathbf {\lambda }$, which signify whether information treatments changed misperceptions. Note that this approach is equivalent to estimating separate models for each information treatment. Note also that we include control variables in vector $\mathbf {D_{it}}$ although we have randomly assigned the treatments to reduce standard errors. Nonetheless, our results are analogous without any control variables.

It is useful to reflect on some potential threats to validity. First, as participants were randomly assigned to different groups, they did not self-select into treatment. Due to random assignment, other events between the two waves are unlikely to have affected the control and the treatment groups differently. Attrition bias may have an impact on our results as more participants made it through the second wave in the control group than in the treatment groups. Nonetheless, Supplementary Table S1 shows that participants in the different groups are similar in observed characteristics, and randomization worked rather well. The only difference that we find is that treatment groups include more men than the control group. However, this is unlikely to influence our results as we control for gender in the empirical model. Finally, the way to measure misperceptions was analogous in both waves, and the control group could not have found out about the experimental treatment. As a result, contamination is unlikely to be an issue in the current setting.

### Supplementary Information

Supplementary Information.

## Data Availability

The data generated and analysed during the current study are available at https://github.com/DeniMazrekaj/Misperceptions-and-Fake-News-Data.
