# Supplementary material for: Identity and inequality misperceptions, demographic determinants and efficacy of corrective measures
Source: Sci Rep. 2024 May 29;14:12300. doi: 10.1038/s41598-024-62046-7 (PMC11136970; doi:10.1038/s41598-024-62046-7)
Supplement: Supplementary file 1 — Supplementary Information. [file 41598_2024_62046_MOESM1_ESM.pdf]

SUPPLEMENTARY INFORMATION

Supplement: UK Version of the Questionnaire

Wave 1

Q1 Were you born in the United Kingdom? Yes / No
Q2 What is your gender? Male / Female
Q3 What is your age?
Q4 What is your gross weekly household income? Less than £400 / £400–£600 / £600–£1.000 / More than £1.000
Q5 Please indicate your marital status. Single / Couple, Married / Separated or Divorced / Widowed
Q6 How many children do you have? I do not have children / 1 / 2 / 3 / 4 / 5 / More than 5
Q7 Which category best describes your highest level of education? Compulsory Education / High School / University (but not finished) / Bachelor's degree / Master Degree / Doctoral Degree
Q8 Which of these descriptions best describes your situation? Please select ONLY one. In paid work / In education / Self-employed / Unemployed and actively looking for a job / Unemployed, wanting a job but not actively looking for a job / Permanently sick or disabled / Retired / In community or military service / Doing housework, looking after children or other persons / Refusal
Q9 Have you ever had a paid job? Yes / No / Refusal/Don't know
Q10 In what year were you last in a paid job?
Q11 In your main job are/were you. . . Please select ONLY one. An employee / Self-employed / Working for your own family's business / Refusal/Don't know
Q12 How many employees (if any) do/did you have?
Q13 Do/did you have a work contract of...Unlimited duration / Limited duration / Do/did you have no contract / Refusal/don't know
Q14 Including yourself, about how many people are/were employed at the place where you usually work/worked?
Q15 In your main job, do/did you have any responsibility for supervising the work of other employees? Yes / No / Refusal/Don't know
Q16 Please indicate on a scale of 0–10 how much the management at your work allows/allowed you to influence policy decisions about the activities of the organization.
Q17 Have you ever been unemployed and seeking work for a period of more than three months in the last five years? Yes / No / Refusal/Don't know
Q18 Have any of these periods lasted for 6 months or more? Yes / No / Refusal/Don't know
Q19 Please consider the total income of all household members. What is the main source of income in your household? Wages or salaries / Income from self-employment / Pensions / Unemployment/redundancy benefit / Any other social benefits or grants / Income from investment, savings, insurance or property / Income from other sources / Refusal/Don't know
Q20 Which of the descriptions comes closest to how you feel about your household's income nowadays? Living comfortably on present income / Coping on present income / Finding it difficult on present income / Finding it very difficult on present income / Refusal/Don't know
Q21 Please indicate on a scale of 0–10 how interested you would say you are in politics.
Q22 Please indicate on a scale of 0–10 how much you would say the political system in the United Kingdom allows people like you to have a say in what the government does.
Q23 Please indicate on a scale of 0–10 how able you think you are to take an active role in a group involved with political issues.
Q24 Please indicate on a scale of 0-.10 how confident you are in your own ability to participate in politics.
Q25 Please indicate on a scale of 0–10 how much you personally trust each of these institutions (0 = Do not trust at all; 10 = Complete trust). Country's parliament / The legal system / The police / Politicians / Political parties / The European Parliament / The United Nations
Q26 Some people don't vote nowadays for one reason or another. Did you vote in the last national election in December 12th, 2019? Yes / No / Refusal/Don't know
Q27 Which party did you vote for in that election? Conservative / Labour / Liberal Democrat / UKIP / Paid Cymru / Green Party / SNP / Brexit Party / Other (write in) / Refusal/Don't know
Q28 Which party do you plan to vote in the next national election? Conservative / Labour / Liberal Democrat / UKIP / Paid Cymru / Green Party / SNP / Brexit Party / Other (write in) / Refusal/Don't know
Q29 In politics people sometimes talk about "left" and "right". Please indicate on a scale of 0-10 where you would place yourself (0 = Left; 10 = Right).
Q30 Please indicate on a scale of 0–10 how religious you think you are (0 = Not religious at all; 10 = Very religious).
Please indicate on a scale of 0–10 whether you agree or disagree with the following statements (0 = Completely disagree; 10 = Completely agree).
Q31 The opinion of ordinary people is worth more than that of experts and politicians.
Q32 Politicians should listen more closely to the problems the people have.
Q33 Ministers should spend less time behind their desks, and more among the ordinary people.
Q34 People who have studied for a long time and have many diplomas do not really know what makes the world go round.
For the next two questions, notice that we consider an ethnic group as a community or population made up of people who share a common cultural background.
Q35 Please indicate on a scale of 0–10 to what extent you think the United Kingdom should allow people of the same race or ethnic group than the majority of the British people to come and live here (0 = Allow none; 10 = Allow many to come and live here).
Q36 Please indicate on a scale of 0–10 to what extent you think the United Kingdom should allow people of the different race or ethnic group than the majority of the British people to come and live here (0 = Allow none; 10 = Allow many to come and live here).
Q37 Please indicate on a scale of 0–10 to what extent you think the United Kingdom should allow people of different religious faith than the majority of the British people to come and live here (0 = Allow none; 10 = Allow many to come and live here).
Q38 Please indicate on a scale of 0–10 to what extent you think the United Kingdom should allow people from poorer countries outside Europe to come and live here (0 = Allow none; 10 = Allow many to come and live here).

Q39 Please indicate on a scale of 0–10 to what extent you think the United Kingdom has become a worse or a better place to live by people coming to live here from other countries (0 = Worse place to live; 10 = Better place to live).

Q40 Typically, how often do you access news? By news we mean national, international, regional/local news and other topical events accessed via radio, TV, newspaper or online. Several times a day / Once a day / Several times a week / Once a week / Several times a month / Once a month / Less often than once a month / Whenever I come across by coincidence / Almost never / Never

Thinking about your news habits, please indicate on a scale of 0–10 how often do you. . . (0 = Never; 10 = Always).

Q41 Read any newspapers in print?

Q42 Listen to news on the radio?

Q43 Watch television news?

Q44 Get news from a social media site (such as Facebook, Twitter, or Snapchat)?

Q45 Get news from a news website or app?

Q46 Which, if any, of the following sources of information do you use to keep up with political issues? Please select all that apply. Friends, relatives or colleagues / National printed newspapers and/or their online sites/apps / Radio broadcasters and/or online sites/apps / TV broadcasters and/or online sites/apps / Politically focused magazines and/or online sites/apps / Political parties and/or their newsletters or online sites / Online specialist sites or political blogs / Social media such as Facebook and Twitter / Don't know / None of these

Q47 Please indicate on a scale of 0-10 how much trust and confidence you have in the mass media – such as newspapers, TV and radio – when it comes to reporting the news fully, accurately and fairly (0 = None at all; 10 = A great deal).

Q48 Please indicate on a scale of 0–10 how much trust and confidence you have in the social media – such as Twitter, Facebook, Instagram and YouTube – when it comes to reporting the news fully, accurately and fairly (0 = None at all; 10 = A great deal).

Q49 Please indicate below whether you get news about politics and current affairs regularly from each of the following sources. For each item, please indicate on a scale of 0–10 if it is something you do regularly (0 = Never; 10 = Always). The Guardian / The Sunday Times / The Times / Daily Mail / The Independent / The Sun / Channel 4 / BBC / ITV / Film 4

Q50 Please indicate on a scale of 0-10 how much trust and confidence you have in the following sources when it comes to reporting the news fully, accurately and fairly (0 = None at all; 10 = A great deal). The Guardian / The Sunday Times / The Times / Daily Mail / The Independent / The Sun / Channel 4 / BBC / ITV / Film 4

Q51 On a typical day, about how much time do you spend using the internet on a computer, tablet, smartphone or other device, whether for work or personal use? Please give your answer in hours and minutes.

Q52 Please indicate on a scale of 0–10 how often you come across news stories about politics online that you think are not fully accurate (0 = Never; 10 = Always).

Q53 Have you ever shared a political news story online that you later found out was made up? Yes / No / No answer

Q54 Have you ever shared a political news story online that you thought at the time was made up? Yes / No / No answer

As you may have heard, there have recently been some instances of so called "fake news stories" circulating widely online. Please indicate on a scale of 0–10 how much responsibility each of the following has in trying to prevent made up stories from gaining attention (0 = No responsibility at all; 10 = A great deal of responsibility).

Q55 Members of the public

Q56 The government, politicians, and elected officials

Q57 Social networking sites like Facebook, Twitter, WhatsApp and search sites like Google

Q58 Media

Q59 Please indicate on a scale of 0–10 how confident you are in your own ability to recognize news that is made up (0 = Not at all confident; 10 = Very confident).

Q60 Please indicate on a scale of 0–10 how much you think these kinds of news stories leave people confused about the basic facts of current issues and events (0 = Not at all; 10 = A great deal).

Please indicate on a scale of 0–10 whether you agree or disagree with the following statements (0 = Completely disagree; 10 = Completely agree).

Q61 There is too much moral decay today.

Q62 The sense of belonging together that we used to have is irrevocably lost.

Q63 Parents no longer adequately educate their children.

Q64 People don't care for each other any more.

Q65 The United Kingdom will face a situation of ever-increasing job insecurity.

Q66 Even more enterprises will move to low-wage countries, threatening employment in the United Kingdom.

Q67 In order to face the competition of other countries we will have to dismantle our welfare state.

Q68 Multinational enterprises will become increasingly powerful, small enterprises are bound to suffer.

Q69 Opening the European frontiers means that our employers will prefer the low-cost workers from poorer countries to our own workers.

Q70 In the future we will become even less open and tolerant with regard to people from other cultures.

Q71 The relationship between Christians and Muslims is bound to become violent in the future.

Q72 The relationship between Christians and Jews is bound to become violent in the future.

Q73 You can generally trust the people who run our government to do what is right.

Q74 For the next question, please consider globalization as the increased trade between countries in goods, services, and investments. Please indicate on a scale of 0–10 whether you think globalization has had a negative or a positive effect on each of the following (0 = Completely negative effect; 10 = Completely positive effect) British factory workers / Multinational corporations based in the United Kingdom / You and your immediate family / The British economy

Please indicate on a scale of 0–10 whether you agree or disagree with the following statements (0 = Completely disagree; 10 = Completely agree).

Q75 It is important to live in secure and safe surroundings.

Q76 People should follow rules at all times, even when no-one is watching.

Q77 It is important that the government is strong and ensures safety against all threats.

Q78 It is important to follow traditions and customs handed down by religion or family.

In the following questions, we refer to legal immigrants as people who were not born in the United Kingdom and legally moved here at a certain point of their life. We are NOT considering irregular migration.

Q79 Think about all of the currently living in the United Kingdom. Out of every 100 people in the United Kingdom, how many are born in another country?

Q80 Fill in the boxes below to indicate how many out of every 100 people in the United Kingdom you think practice each religion. Christianity / Islam / Buddhism / Hinduism / Other Religions/Atheist/No religious affiliation

Q81 Out of every 100 people, who are between 20 and 64 years old, in the United Kingdom how many are currently unemployed? By unemployed we mean people who are currently not working but searching for a job (and maybe unable to find one). Now let's compare this to the number of unemployed among foreign-born people. Out of every 100 foreign-born people how many do you think are currently unemployed?

Q82 The poverty line is the estimated minimum level of income needed to secure the necessities of life. Out of every 100 adult people born in the United Kingdom, how many live below the poverty line? Let's compare this to poverty among legal immigrants. Out of every 100 legal immigrants in the United Kingdom today, how many do you think live below the poverty line?

Q83 The International Organization for Migration (IOM) defines irregular migration as "movement that takes place outside the regulatory norms of the sending, transit and receiving country". A migrant in an irregular situation may fall within one or more of the following circumstances: He or she may enter the country irregularly; he or she may reside in the country irregularly; he or she may be employed in the country irregularly. Think about the evolution of the irregular migration flows in Europe in the last 3 years. It has increased over time / It has decreased over time / It has kept constant over time / Don´t know

Q84 Think about the evolution of detections of illegal border crossing at the EU's external borders in the last 3 years. It has increased over time / It has decreased over time / It has kept constant over time / Don´t know

Q85 How many Islamist terrorists do you think have been arrested in the United Kingdom in 2018?

Q86 How many people do you think have been killed during terror attacks committed by Islamist terrorists in the United Kingdom in the last 5 years?

Q87 Please indicate on a scale of 0–10 whether you agree or disagree with the following statement (0 = Completely disagree; 10 = Completely agree): Most crimes in the UK are committed by foreigners.

Q88 What percentage of the prison population in the United Kingdom are foreign national prisoners?

Q89 What do you think is the income share of the poorest 20% of all people living in the United Kingdom?

Q90 What do you think is the income share of the richest 10% of all people living in the United Kingdom?

Q91 How large is the share of taxes and social contributions in percentage of GDP in the United Kingdom?

Q92 According to the share of taxes and social contributions as a percentage of GDP, in which position do you think the United Kingdom is among the 28 Union European countries? Notice that a higher position in the list implies a larger share.

Q93 Please consider corruption in a broad sense, including offering, giving, requesting and accepting bribes or kickbacks, valuable gifts and important favors, as well as any abuse of power for private gain. Transparency International is the leading global civil organization on the fight against corruption. Each year they elaborate a Corruption Perceptions Index which ranks 180 countries and territories by their perceived levels of public sector corruption according to experts and business people. In which position do you think the United Kingdom is among the 28 Union European countries?

Q94 There are people who tend to be towards the top of our society and people who tend to be towards the bottom. Below is a scale that runs from top to bottom. On a scale of 1–10 Where you would put yourself (1 = Bottom of our society; 10 = Top of our society).

Please indicate on a scale of 0–10 to what extent you agree with the following statements (0 = Completely disagree; 10 = Completely agree).

Q95 I experience a general sense of emptiness.

Q96 There are many people I can trust completely.

Q97 I miss having people around me.

Q98 I often feel rejected.

Q99 I have enough opportunities to advance in life

Q100 I know exactly where I feel at home and where I belong.

Wave 2

New questions are marked with "N" after the number, the information treatment is marked with "T" after the number

Q1 Were you born in the United Kingdom? Yes / No

Q2 What is your gender? Male / Female / Non-Binary

Q3 What is your age?

Q3–N How long have you lived in your current city? (years)

Q4 What is your gross weekly household income? Less than £400 / £400–£600 / £600–£1.000 / More than £1.000

Q5 Please indicate your marital status. Single / Couple, Married / Separated or Divorced / Widowed

Q6 How many children do you have? I do not have children / 1 / 2 / 3 / 4 / 5 / More than 5

Q7 Which category best describes your highest level of education? Compulsory Education / High School / University (but not finished) / Bachelor Degree / Master Degree / Doctoral Degree

Q7–T1 In the last survey, you estimated population share of immigrants as .... In 2019, population share of immigrants in the UK was 14%.

Q7–T2 In the last survey, you estimated income share of the richest 10% as .... In 2019, income share of the richest 10% in the UK was 26%.

Q8 Which of these descriptions best describes your situation? Please select ONLY one. In paid work / In education / Self employed / Unemployed and actively looking for a job / Unemployed, wanting a job but not actively looking for a job / Permanently sick or disabled / Retired / In community or military service / Doing housework, looking after children or other persons / Refusal/Don't know

Q9 Have you ever had a paid job? Yes / No / Refusal/Don't know

Q10 In what year were you last in a paid job? Write in year / Refusal/Don't know

Q11 In your main job are/were you. . . Please select ONLY one. An employee / Self-employed / Working for your own family's business / Refusal/Don't know

Q12 How many employees (if any) do/did you have? Write in the number of employees / Refusal/Don't know

Q13 Do/did you have a work contract of...Unlimited duration / Limited duration / Do/did you have no contract? / Refusal/Don't know

Q14 Including yourself, about how many people are/were employed at the place where you usually work/worked...Under 10 / 10 to 24 / 25 to 99 / 100 to 499 / 500 or more / Refusal/Don't know

Q17 Have you ever been unemployed and seeking work for a period of more than three months in the last five years? Yes / No / Refusal/Don't know

Q18 Have any of these periods lasted for 6 months or more? Yes / No / Refusal/Don't know

Q19 Please consider the total income of all household members. What is the main source of income in your household? Wages or salaries / Income from self-employment / Pensions / Unemployment/redundancy benefit / Any other social benefits or grants / Income from investment, savings, insurance or property / Income from other sources / Refusal/Don't know

Q20 Which of the descriptions comes closest to how you feel about your household's income nowadays? Living comfortably on present income / Coping on present income / Finding it difficult on present income / Finding it very difficult on present income / Refusal/Don't know

Q21 Please indicate on a scale of 0–10 how interested you would say you are in politics (0 = Not at all interested; 10 = Very interested).

Q22 Please indicate on a scale of 0–10 how much you would say the political system in the United Kingdom allows people like you to have a say in what the government does (0 = Not at all; 10 = A great deal).

Q25 Please indicate on a scale of 0–10 how much you personally trust each of these institutions (0 = Do not trust at all; 10 = Complete trust). Country's parliament / The legal system / The police / Politicians / Political parties / The European Parliament / The United Nations

Q26 Some people don't vote nowadays for one reason or another. Did you vote in the last national election in December 12th, 2019? Yes / No / Refusal/Don't know

Q27 Which party did you vote for in that election? Conservative / Labour / Liberal Democrat / UKIP / Paid Cymru / Green Party / SNP / Brexit Party / Other (write in) / Refusal/Don't know

Q28 Which party do you plan to vote in the next national election? Conservative / Labour / Liberal Democrat / UKIP / Paid Cymru / Green Party / SNP / Brexit Party / Other (write in) / Refusal/Don't know

Q29 In politics people sometimes talk about "left" and "right". Please indicate on a scale of 0–10 where you would place yourself (0 = Left; 10 = Right).

Q30 Please indicate on a scale of 0–10 how religious you think you are (0 = Not religious at all; 10 = Very religious).

Please indicate on a scale of 0–10 whether you agree or disagree with the following statements (0 = Completely disagree; 10 = Completely agree).

Q31 The opinion of ordinary people is worth more than that of experts and politicians.

Q32 Politicians should listen more closely to the problems the people have.

Q33 Ministers should spend less time behind their desks, and more among the ordinary people.

Q34 People who have studied for a long time and have many diplomas do not really know what makes the world go round.

For the next two questions, notice that we consider an ethnic group as a community or population made up of people who share a common cultural background.

Q35 Please indicate on a scale of 0–10 to what extent you think the United Kingdom should allow people of the same race or ethnic group than the majority of the British people to come and live here (0 = Allow none; 10 = Allow many to come and live here).

Q36 Please indicate on a scale of 0–10 to what extent you think the United Kingdom should allow people of the different race or ethnic group than the majority of the British people to come and live here (0 = Allow none; 10 = Allow many to come and live here).

Q37 Please indicate on a scale of 0–10 to what extent you think the United Kingdom should allow people of different religious faith than the majority of the British people to come and live here (0 = Allow none; 10 = Allow many to come and live here).

Q38 Please indicate on a scale of 0–10 to what extent you think the United Kingdom should allow people from poorer countries outside Europe to come and live here (0 = Allow none; 10 = Allow many to come and live here).

Q39 Please indicate on a scale of 0–10 to what extent you think the United Kingdom has become a worse or a better place to live by people coming to live here from other countries (0 = Worse place to live; 10 = Better place to live).

Q40 Typically, how often do you access news? By news we mean national, international, regional/local news and other topical events accessed via radio, TV, newspaper or online. Several times a day / Once a day / Several times a week / Once a week / Several times a month / Once a month / Less often than once a month / Whenever I come across by coincidence / Almost never / Never

Thinking about your news habits, please indicate on a scale of 0-10 how often do you. . . (0 = Never; 10 = Always).

Q41 Read any newspapers in print?

Q42 Listen to news on the radio?

Q43 Watch television news?

Q44 Get news from a social media site (such as Facebook, Twitter, or Snapchat)?

Q45 Get news from a news website or app?

Q46 Which, if any, of the following sources of information do you use to keep up with political issues? Please select all that apply. Friends, relatives or colleagues / National printed newspapers and/or their online sites/apps / Radio broadcasters and/or online sites/apps / TV broadcasters and/or online sites/apps / Politically focused magazines and/or online sites/apps / Political parties and/or their newsletters or online sites / Online specialist sites or political blogs / Social media such as Facebook and Twitter / Don't know / None of these

Q47 Please indicate on a scale of 0-10 how much trust and confidence you have in the mass media – such as newspapers, TV and radio – when it comes to reporting the news fully, accurately and fairly (0 = None at all; 10 = A great deal).

Q48 Please indicate on a scale of 0–10 how much trust and confidence you have in the social media – such as Twitter, Facebook, Instagram and YouTube – when it comes to reporting the news fully, accurately and fairly (0 = None at all; 10 = A great deal).

Q51 On a typical day, about how much time do you spend using the internet on a computer, tablet, smartphone or other device, whether for work or personal use? Please give your answer in hours and minutes.

Q52 Please indicate on a scale of 0–10 how often you come across news stories about politics online that you think are not fully accurate (0 = Never; 10 = Always).

Q53 Have you ever shared a political news story online that you later found out was made up? Yes / No / No answer

Q54 Have you ever shared a political news story online that you thought at the time was made up? Yes / No / No answer

Q55–N To the best of your knowledge, how likely is it that the claim in each of the below headlines is correct? (Extremely unlikely / Somewhat unlikely / Neither likely nor unlikely / Somewhat likely / Extremely likely)
Pope Francis Shocks World, Endorses Donald Trump for President / Israeli Defense Minister: If Pakistan send group troops to Syria on any pretext, we will destroy this country with a nuclear attack / Macron allowed the use of Sputnik V vaccine in France / Italian town forbids Christmas carols not to insult migrants / Ukraine will buy the Russian vaccine from Germany at an inflated price / Donald Trump nominated for the 2021 Nobel Peace Prize / Amazon had sales income of €44bn in Europe in 2020 but paid no corporation tax / French homeless population doubled since 2012 / Switzerland ends talks with EU on co-operation agreement / Iowa workers fired for refusing COVID vaccine still eligible for unemployment benefits

Q56–N Would you consider sharing each of the following stories online (for example through Facebook or Twitter)? (No, Maybe, Yes)
Pope Francis Shocks World, Endorses Donald Trump for President / Israeli Defense Minister: If Pakistan send group troops to Syria on any pretext, we will destroy this country with a nuclear attack / Macron allowed the use of Sputnik V vaccine in France / Italian town forbids Christmas carols not to insult migrants / Ukraine will buy the Russian vaccine from Germany at an inflated price / Donald Trump nominated for the 2021 Nobel Peace Prize / Amazon had sales income of €44bn in Europe in 2020 but paid no corporation tax / French homeless population doubled since 2012 / Switzerland ends talks with EU on co-operation agreement / Iowa workers fired for refusing COVID vaccine still eligible for unemployment benefits

Q59 Please indicate on a scale of 0–10 how confident you are in your own ability to recognize news that is made up (0 = Not at all confident; 10 = Very confident).

Q60 Please indicate on a scale of 0–10 how much you think these kinds of news stories leave people confused about the basic facts of current issues and events (0 = Not at all; 10 = A great deal).
Please indicate on a scale of 0–10 whether you agree or disagree with the following statements (0 = Completely disagree; 10 = Completely agree).

Q61 There is too much moral decay today.

Q62 The sense of belonging together that we used to have is irrevocably lost.

Q63 Parents no longer adequately educate their children.

Q64 People don't care for each other any more.

Q65 The United Kingdom will face a situation of ever-increasing job insecurity.

Q66 Even more enterprises will move to low-wage countries, threatening employment in the United Kingdom.

Q67 In order to face the competition of other countries we will have to dismantle our welfare state.

Q68 Multinational enterprises will become increasingly powerful, small enterprises are bound to suffer.

Q69 Opening the European frontiers means that our employers will prefer the low-cost workers from poorer countries to our own workers.

Q70 In the future we will become even less open and tolerant with regard to people from other cultures.

Q71 The relationship between Christians and Muslims is bound to become violent in the future.

Q72 The relationship between Christians and Jews is bound to become violent in the future.

Q73 Please indicate on a scale of 0–10 whether you agree or disagree with the following statement (0 = Completely disagree; 10 = completely agree). You can generally trust the people who run our government to do what is right.

Q74 For the next question, please consider globalization as the increased trade between countries in goods, services, and investments. Please indicate on a scale of 0–10 whether you think globalization has had a negative or a positive effect on each of the following (0 = Completely negative effect; 10 = Completely positive effect). British factory workers / Multinational corporations based in the United Kingdom / You and your immediate family / The British economy
Please indicate on a scale of 0–10 whether you agree or disagree with the following statements (0 = Completely disagree; 10 = Completely agree).

Q75 It is important to live in secure and safe surroundings.

Q76 People should follow rules at all times, even when no-one is watching.

Q77 It is important that the government is strong and ensures safety against all threats.

Q78 It is important to follow traditions and customs handed down by religion or family.
In the following four questions, we refer to legal immigrants as people who were not born in the United Kingdom and legally moved here at a certain point of their life. We are NOT considering irregular migration.

Q79 Think about all of the currently living in the United Kingdom. Out of every 100 people in the United Kingdom, how many are born in another country?

Q80 Fill in the boxes below to indicate how many out of every 100 people in the United Kingdom you think practice each religion. Christianity / Islam / Buddhism / Hinduism / Other Religions/Atheist/No religious affiliation

Q81 Out of every 100 people, who are between 20 and 64 years old, in the United Kingdom how many are currently unemployed? By unemployed we mean people who are currently not working but searching for a job (and maybe unable to find one). Now let's compare this to the number of unemployed among foreign-born people. Out of every 100 foreign-born people how many do you think are currently unemployed?

Q82 The poverty line is the estimated minimum level of income needed to secure the necessities of life. Out of every 100 adult people born in the United Kingdom, how many live below the poverty line? Let's compare this to poverty among legal immigrants. Out of every 100 legal immigrants in the United Kingdom today, how many do you think live below the poverty line?

Q83 The International Organization for Migration (IOM) defines irregular migration as "movement that takes place outside the regulatory norms of the sending, transit and receiving country". A migrant in an irregular situation may fall within one or more of the following circumstances: He or she may enter the country irregularly; he or she may reside in the country irregularly; he or she may be employed in the country irregularly. Think about the evolution of the irregular migration flows in Europe in the last 3 years. It has increased over time / It has decreased over time / It has kept constant over time / Don´t know

Q84 Think about the evolution of detections of illegal border crossing at the EU's external borders in the last 3 years. It has increased over time / It has decreased over time / It has kept constant over time / Don´t know

Q85 How many Islamist terrorists do you think have been arrested in the United Kingdom in 2020?

Q86 How many people do you think have been killed during terror attacks committed by Islamist terrorists in the United Kingdom in the last 5 years?

Q87 Please indicate on a scale of 0–10 whether you agree or disagree with the following statement (0 = Completely disagree; 10 = Completely agree). Most crimes in the UK are committed by foreigners.

Q88 What percentage of the prison population in the United Kingdom are foreign national prisoners?

Q89 What do you think is the income share of the poorest 20% of all people living in the United Kingdom?

Q90 What do you think is the income share of the richest 10% of all people living in the United Kingdom?

Q91 How large is the share of taxes and social contributions in percentage of GDP (Gross Domestic Product) in the United Kingdom?

Q92 According to the share of taxes and social contributions as a percentage of GDP, in which position do you think the United Kingdom is among the 27 European Union countries and the UK (28 countries in total)? Notice that a higher position in the list implies a larger share.

For the next questions, please consider corruption in a broad sense, including offering, giving, requesting and accepting bribes or kickbacks, valuable gifts and important favors, as well as any abuse of power for private gain.

Q93 Transparency International is the leading global civil organization on the fight against corruption. Each year they elaborate a Corruption Perceptions Index which ranks 180 countries and territories by their perceived levels of public sector corruption according to experts and business people. In which position do you think the United Kingdom is among the 27 European Union countries and the UK (28 countries in total)?

Q94 There are people who tend to be towards the top of our society and people who tend to be towards the bottom. On a scale of 0–10 Where you would put yourself (0 = Bottom of our society; 10 = Top of our society).

Please indicate on a scale of 0–10 to what extent you agree with the following statements (0 = Completely disagree; 10 = Completely agree).

Q95 I experience a general sense of emptiness.

Q96 There are many people I can trust completely.

Q97 I miss having people around me.

Q98 I often feel rejected.

Q99 I have enough opportunities to advance in life.

Q100 I know exactly where I feel at home and where I belong.

Supplement: Definitions and Data Sources of Actual Statistics

To compare the perceptions of our respondents with actual statistics, we used the following definitions and data sources.

Foreign Born

For the question ”Out of every 100 people in [country], how many are born in another country?”, we used the share of foreign-born in the entire population. Data are taken from Eurostat, Population and Migration Statistics, code: migr_pop3ctb. We employed the most recent data available at the time of the survey, which were the population figures for 2018. The share of foreign born amounts to 17% in Germany and 14% in the UK.

Muslim Population

The respondents' estimate for the Muslim share was captured by the question ”How many out of every 100 people in [country] you think practice each religion.” There is no uniform database to cover the actual share of people practicing Islam. In Germany, the share of the Muslim population is 5.1%; the estimate refers to 2018 and is taken from *Forschungsgruppe Weltanschauungen in Deutschland* (https://fowid.de/meldung/religionszugehoerigkeiten-2018). For the UK, we relied on the CIA World Factbook (https://www.cia.gov/the-world-factbook/countries/); the most recent estimate is from 2011 with a value of 4.4%.

Poverty

Respondents were asked ”The poverty line is the estimated minimum level of income needed to secure the necessities of life. Out of every 100 adult people born in [country], how many live below the poverty line?”. Eurostat sets this threshold of being at risk of poverty at 60% of median equivalised income after social transfers. We used the poverty rate of the population aged 18 and over from Eurostat (Quality of life, code: ilc_li31). In March 2020, the most recent data were available for the years 2018 (Germany) and 2017 (UK). The poverty rates were 16% in Germany and 16% in the UK.

Income Share of the Top Decile

We elicited the respondents' estimates for the income share of the top 10% earners by asking: ”What do you think is the income share of the richest 10% of all people living in [country]?” We contrast these estimates with data from Eurostat (Quality of life, code: ilc_di01), which provides the distribution of incomes by quantiles. The income shares for 2018 (available in March 2020) were 26% in Germany and the UK.

Supplementary Table S1. Descriptive statistics ($N = 3,072$)

	No treatment	Immigration treatment	Income treatment
Gender	.537	.426	.415
Age			
18-35 y.o.	.140	.121	.113
36-54 y.o.	.438	.435	.447
55-70 y.o.	.421	.444	.440
Education	.389	.435	.427
Marital Status	.398	.426	.378
Household Income			
Low income	.213	.186	.200
Middle income	.596	.594	.599
High income	.191	.220	.201
Labour-Market Position			
Employed	.742	.765	.759
Unemployed	.019	.016	.015
Out of labor force	.239	.219	.227
Political Orientation			
Left	.203	.201	.230
Center	.629	.638	.581
Right	.169	.160	.189
Observations	1,303	874	895

Notes: The numbers in the table represent the proportions.
